# Shall I Trust You? From Child–Robot Interaction to Trusting Relationships

**DOI:** 10.3389/fpsyg.2020.00469

**Published:** 2020-04-03

**Authors:** Cinzia Di Dio, Federico Manzi, Giulia Peretti, Angelo Cangelosi, Paul L. Harris, Davide Massaro, Antonella Marchetti

**Affiliations:** ^1^Research Unit on Theory of Mind, Department of Psychology, Università Cattolica del Sacro Cuore, Milan, Italy; ^2^School of Computer Science, The University of Manchester, Manchester, United Kingdom; ^3^Graduate School of Education, Harvard University, Cambridge, MA, United States

**Keywords:** developmental robotics, HRI, Theory of Mind, attachment, social interaction

## Abstract

Studying trust in the context of human–robot interaction is of great importance given the increasing relevance and presence of robotic agents in the social sphere, including educational and clinical. We investigated the acquisition, loss, and restoration of trust when preschool and school-age children played with either a human or a humanoid robot *in vivo*. The relationship between trust and the representation of the quality of attachment relationships, Theory of Mind, and executive function skills was also investigated. Additionally, to outline children’s beliefs about the mental competencies of the robot, we further evaluated the attribution of mental states to the interactive agent. In general, no substantial differences were found in children’s trust in the play partner as a function of agency (human or robot). Nevertheless, 3-year-olds showed a trend toward trusting the human more than the robot, as opposed to 7-year-olds, who displayed the reverse pattern. These findings align with results showing that, for 3- and 7-year-olds, the cognitive ability to switch was significantly associated with trust restoration in the human and the robot, respectively. Additionally, supporting previous findings, we found a dichotomy between attributions of mental states to the human and robot and children’s behavior: while attributing to the robot significantly lower mental states than the human, in the Trusting Game, children behaved in a similar way when they related to the human and the robot. Altogether, the results of this study highlight that similar psychological mechanisms are at play when children are to establish a novel trustful relationship with a human and robot partner. Furthermore, the findings shed light on the interplay – during development – between children’s quality of attachment relationships and the development of a Theory of Mind, which act differently on trust dynamics as a function of the children’s age as well as the interactive partner’s nature (human vs. robot).

## Introduction

One of the challenges of contemporary robotics is *long-term interaction*, which assumes that competent robot partners will have many human-like characteristics, enabling the complexity and multidimensionality of human interactions. This objective has been strengthened by a new interdisciplinary approach to robotics, i.e. Developmental Robotics (Cangelosi and Schlesinger, 2015). For example, Vinanzi et al. (2019) have proposed an artificial cognitive architecture to simulate human decision making in the robot by using concepts from developmental theories, such as Theory of Mind (ToM). From this perspective, the implementation of an artificial architecture, together with an understanding of the human’s response to the behavior of a robot within a relational context, aims to shed light on the processes involved in establishing a relationship with robotic agents (e.g. Wykowska et al., 2016; Wiese et al., 2017). Within this framework, trust comes into play as a key psychological component underpinning successful interpersonal relationships, particularly when these include at least one robotic agent. In the present study, we observed children between the ages of 3 and 9 who established relationships of trust with a human or the humanoid robot NAO in a simple “guessing” game in which the child and the human or robot played together. Furthermore, not only did we assess trust acquisition, but also a key feature of real-life relational dynamics: trust restoration after trust loss. As a matter of fact, trust is a dynamic process based on past relational experiences and, as such, it is subject to fluctuations operationalized in this study via three phases of trust: acquisition, loss, and restoration. The latter phase is of particular interest. While human forgiveness has been studied in different conditions (see, for example, Grover et al., 2019), the investigation of how relational failures may affect trust restoration in a relationship with a robot is still unexplored.

In psychology, trust can be described as “a multidimensional psychological attitude involving beliefs and expectations about the reliability of the trustee resulting from social experiences involving uncertainty and risk” (Jones and George, 1998; in Lewis et al., 2018, p. 137). Trust in the choices of unknown people can be envisaged also in situations where we passively witness their behavior, with consequences on our own decisions (e.g. Rizzato et al., 2016). The multidimensional nature of trust encompasses the idea that trust can be built based on either (or both) objective factors or (and) an emotional, quite irrational, attitude toward the partner (Lahno, 2001). In this light, emotional trust can be conceived as somewhat independent of objective information. In this study, we recreated a situation of total uncertainty in which the choices of a partner, who should be trusted, are not based on the evaluation of objective elements, and also the decision of the child to trust in the partner are devoid of rational elements. Rather, the decision to trust or not to trust the partner’s choices is consequentialist in nature considering that, until proven otherwise, the partner is always accurate in her/his/its choices. That is, trust is progressively built through constant endorsement of the play partners’ reliability in providing correct responses (see, Rotenberg, 2010). From this perspective, conformation to the other’s choices reflected levels of trust acquisition as well as acceptance of the other as a potential partner (Nass et al., 1995; Nass and Moon, 2000).

The establishment of trusting relationships is critical for effective interpersonal dynamics. This is particularly relevant where children are called to build new relationships with peers, educators, and other adults. An example of the importance of the construction of interpersonal trust is highlighted in a study with children under protection services (Petrocchi et al., 2018). In these critical circumstances, not only does the success of the social interventions rely on building trusting relationships between the child’s parents and the social workers, but also between the latter and the child in need of psychosocial adjustment (Hafford-Letchfield and Spatcher, 2007). Developmental research on the construction of trusting relationships shows that trust dynamis change significantly as a function of age. For example, children aged 3 years tend to display trust if the informant is consistently 182 accurate (Clément et al., 2004; Koenig et al., 2004; Pasquini et al., 2007) but are relatively unforgiving in case of mistakes (Harris, 2007), effectively showing a certain behavioral rigidity. With development, particularly from 4 years of age, children become more flexible: they do not rely on another’s testimony in an indiscriminate fashion (Harris, 2007) and show selective trust in others’ testimony (Clément et al., 2004; Chan and Tardif, 2013). They attend both to the information available at that moment, and to the reliability that a person has shown in the past.

Human trusting relationships are also shaped by past relational histories, originating with primary caregivers (e.g. Camisasca et al., 2017; Giovanelli et al., 2020; Marchetti et al., 2020) and extending to subsequent, significant affective relationships (Bowlby, 1969, 1973, 1980). It has been suggested that children’s decision to place trust in an unknown informant, especially in a context of uncertainty, may also depend on generalizing from their personal attachment history (Fonagy, 1998; Allison and Fonagy, 2016; Fonagy et al., 2019; see also, Bo et al., 2017; Luo et al., 2018). For example, securely attached children are more flexible in establishing trustful relationships with epistemically reliable strangers than children with a fragile relational past (see, for example, Corriveau et al., 2009). In this view, we may ask about interactions that involve partners with whom there is no affective history and with whom a relationship needs to be built on the basis of novel interactional dynamics that develop *hic et nunc*.

Likewise, the development of the individual’s cognitive competencies is important, particularly for the definition of the informant’s epistemic reliability. Cognitive skills allow individuals to reason about the other’s perspective and to objectively evaluate informational access. In this respect, the development of a ToM enabling individuals to conceptualize the mental states that guide behavior (Wimmer and Perner, 1983) and social competence (Premack and Woodruff, 1978; Perner and Wimmer, 1985; see also, Lombardi et al., 2018; for a review, see Wellman et al., 2001) is a necessary prerequisite for the establishment of trusting relationships (Fusaro and Harris, 2008; Lecciso et al., 2011; Sharp et al., 2011; Lucas et al., 2013; Brosseau-Liard et al., 2015; Rotenberg et al., 2015; Van Reet, 2015). The association between the establishment of trust and the development of ToM competencies was first hypothesized by Koenig and Harris (2005) who found that only 4-year-olds, and not 3-year-olds, showed selective trust toward a previously accurate informant. More recently, associating trust beliefs with ToM abilities in children aged 9 years, Rotenberg et al. (2015) further showed that children’s trust beliefs in others are associated with both second-order false belief ToM ability as well as with advanced ToM abilities (see also Van Reet et al., 2015). As well-documented (e.g. Carlson and Moses, 2001; Frye et al., 1995), there is a strict relationship between false belief understanding and more general executive function skills. One may then question about the overlap between these competencies in building trust. Still, socio-cognitive skills mediated by one’s ability to understand the others’ knowledge, like false belief, appear to be more influential in building selective trust rather than more general executive function skills, at least in some cultures (Lucas et al., 2013).

In relation to human–robot interaction, studies that have specifically investigated trust in a robot agent or system have typically involved adult participants. These studies have either used explicit measures of trust assessment, mostly involving self-reports (e.g. Yagoda and Gillan, 2012), or implicit measures of trust assessment. Explicit measures of trust are strongly subject to the idiosyncratic attitude and the impression that one has of the robot, which are often based on beliefs and not on actual interactional experiences with the robot; on the other hand, implicit measures of trust generally involve the postulation of hypotheses framed by specific environmental and theoretical conditions that are then tested during actual interaction with a robotic system. Gaudiello et al. (2016), for example, investigated the role played by functional acceptance (perceived ease of use, usefulness) and social acceptance (generally linked to social competencies) of the robot iCub for effective human–robot interaction. These two aspects appear to be most consistently associated with an enduring perception of the robot’s skills, i.e. its usefulness and sociality (Shaw-Garlock, 2009; Heerink, 2010). As a most comprehensive measure of functional and social acceptance of the robot, the users’ trust in the robot was assessed as a function of the robot’s social and functional knowledge. The users’ trust in the robot prevalently relied on its functional rather than social knowledge, although data generally highlighted adults’ poor acceptance of, and a predominant distrust in robots. With children, the factors underpinning child human–robot interaction have not been systematically explored. There are several studies that inform about ways in which children interact, play, and learn from a robotic agent in school and educational contexts (Kanda et al., 2004; Okumura et al., 2013a, b; Breazeal et al., 2016; Baxter et al., 2017; Belpaeme et al., 2018; Cangelosi and Schlesinger, 2018; Di Dio et al., 2019). These studies have shown that children tend to interact with robot partners in a human-like manner, proving to be sensitive to verbal and non-verbal signals, such as eye gaze (Okumura et al., 2013a, b), and often attributing mentalistic competencies to the robot (for a review, see Marchetti et al., 2018). In this respect, the work by Short et al. (2010) shows that unfair/cheating robots in a common “rock-paper-scissors” child-game are able to elicit interest in the child as well as a greater tendency to attribute intentions to the robot. This study brings further support to the idea that human-like behavior (either trustful or even deceptive) is associated to a greater interactional potential toward a robot partner.

In the present study, trust was explored through a novel Trusting Game (TG) named “Guess where it is” requiring the interactive partner (either the human or the robot) and, subsequently, the child to guess the position of a doll hidden under a box. Through the structure of the game, we set the conditions for the child to consequentially make the same decisions as the play partner, thus ultimately establishing a trusting relationship (e.g. Nass and Moon, 2000): the other becomes trustworthy because it demonstrates that her/his/its choices, even if random, always lead to a correct answer. This procedure benefitted from having the child gradually build trust in the partner during a social interaction. It was chosen not to establish epistemic trust before the game following best known procedures (see, for example, Koenig and Harris, 2005; Corriveau and Harris, 2009) because we also wanted to appreciate the *dynamics* of trust construction when interacting with different relational agents, i.e. the human and the robot. Once trust had been acquired, as indexed by a consistent agreement between the play partner’s and the child’s responses, the phases of loss of trust and trust restoration put the child’s trust to test. These latter phases were most critical for the child because s/he had to reconsider the newly established trust in the robot or the human. To better understand what psychological factors are in place when building a trusting relationship with the robot, as compared to the human, we addressed specific different chronological ages (e.g. Lombardi et al., 2017) where the development of affective and cognitive processes may be distinctively influential on trust. Also, to better appreciate how trust is configured within robot–human and human–human interaction, we avoided creating competitive or collaborative conditions that could have polarized the dynamics of trust-building. As a matter of fact, the type of interaction can significantly influence trust (Hancock et al., 2011) by negatively or positively skewing trust in case of competition or collaboration, respectively (Kidd, 2003; Kuchenbrandt and Eyssel, 2012). Therefore, we had the children play for the mere fun of playing with a little thank-you gift delivered at the end of the game (the structure of the TG is detailed in section “Materials and Methods”). Finally, we further assessed the distinctive contribution of ToM and executive function skills in building trust at different developmental ages, thus extending current literature by also exploring these cognitive components when children interacted with a robot or a human agent.

To make the child perceive the robotic agent NAO as a real interactional partner, it was introduced to children in a preliminary session when they were familiarized with some of the robot’s physical and social skills (walking, moving its arms, talking, greeting, etc.) (see Vogt et al., 2017). To make its behavior human-like, when playing its turn during the TG, NAO used simple and clear verbal indications, accompanied by gestures indicating the possible target position of the doll. Additionally, the robot was programmed to alternate between looking at the play setup and the child, reproducing a realistic attentional shift (Zanatto et al., 2019).

The children’s perception of the robot’s mental qualities as compared to the human was evaluated through the Attribution of Mental States (AMS) questionnaire (Di Dio et al., 2018, 2019). This measure has consistently shown that school-age children do discriminate between the human and the robot in mental terms, although, during interaction, children also typically display similar behaviors toward both. Accordingly, we hypothesized to find substantial differences in the children’s attribution of mental states to the human and the robot, whereas a similar trust-building dynamics when interacting with either partner during the TG. Additionally, we hypothesized to find a greater tendency to trust, especially in the human, among younger children whose trust is possibly mainly driven by affect rather than cognition. On the other hand, we hypothesized to find the establishment of more reflective trusting relationships among children given the development of ToM competencies. No specific predictions were advanced with respect to the role of executive functions in trust dynamics given the fair lack of specific evidence in this respect.

## Materials and Methods

### Participants

Ninety-four (94) Italian kindergarten and school-age children participated in the experiment. The children were divided into four age groups as follows: 3-year-olds (*N* = 22, 9 females), 5-year-olds (*N* = 24, 13 females), 7-year-olds (*N* = 25, 13 females), and 9-year-olds (*N* = 23, 12 females). The children were recruited from a preschool and a primary school of Milan. The children’s parents received a written explanation of the procedure of the study, the measurement items, and the materials used, and they gave written consent. Children were not identified by parents or teachers for learning and/or socio-relational difficulties. The study was approved by the Local Ethics Committee (Università Cattolica del Sacro Cuore, Milan).

### Tasks

The children were assessed in two experimental sessions on different days within a 2-week time frame. In the first session, the children were administered the following tests: AMS scale (inspired by the work of Martini et al., 2016), TG task [inspired by the work of Yang et al. (2017)], and a first-order and (for 5- to 9-year-olds) a second-order False-Belief task (Wimmer and Perner, 1983; Perner and Wimmer, 1985). In the second session, the children were administered a further version of the first-order and second-order False-Belief task, the quality of attachment relationships (SAT) test (Liverta Sempio et al., 2001), an executive function task (Dimensional Change Card Sort, DCCS; Zelazo, 2006) for the 3- and 5-year-olds, and the Developmental NEuroPSYchological Assessment (NEPSY II; Korkman et al., 2007) subtest for the 7- and 9-year-olds. Both tests assess the ability to switch between responses.

#### Attribution of Mental States

The AMS scale is a measure of the mental states that participants attribute when looking at pictures depicting specific characters, in this case a human and the robot NAO. The scale is an *ad hoc* questionnaire that was based on Martini et al. (2016). AMS has been used in previous works (Di Dio et al., 2018, 2019; see also, Di Dio et al., 2020; Manzi et al., 2020) and has proven fairly consistent in outlining age-specific response patterns with respect to attribution of mental states to both robots and humans. Children were asked 25 questions grouped in five different state categories: Perceptive, Emotional, Desires and Intentions, Imaginative, and Epistemic. The child had to respond “Yes” or “No” to each question. If the answer was Yes, then the experimenter asked a follow-up question: “How much? A little bit or very much?”, yielding a 3-point scale. For example, in answer to the question: “Do you think that he/she/it can understand?”, the range of answers could be: No (0), Yes, a little bit (1), or Yes, very much (2). The total score was the sum of all answers (range = 0–50); the five partial scores were the sum of the answers within each category (score range = 0–10).

#### Trusting Game

**FIGURE 1:**
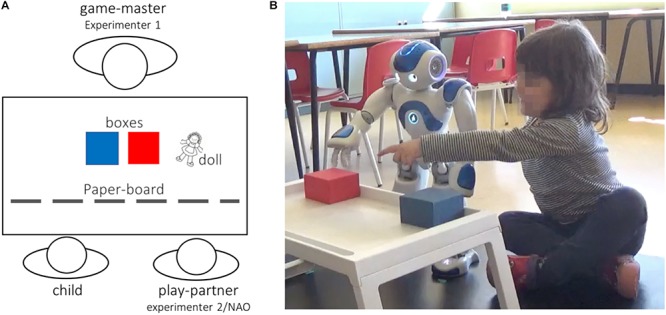
Overview of the experimental setup in the Trust Game. **(A)** Overview of the participants’ seating arrangement and stimuli. **(B)** Photograph representing the SoftBank Robotics NAO humanoid robot while playing the Trust Game with a child (subject).

The TG was inspired by the work of Yang et al. (2017). The game requires the play partner (either the human or the robot) and the child guess the position of a doll hidden under a box. By its nature, the game is neither explicitly collaborative nor competitive since both players have to independently guess the position of the doll and correct guesses do not lead to any tangible reward. The TG involves two players (i.e. a child – participant – playing with either the experimenter or the robot) and a game-master (i.e. a second experimenter). The game consists of presenting to the players two boxes and a little doll that are positioned on a table that looks very much like a coffee table and at which both players are seated. The game-master, who sits on the opposite side of the table, hides the doll under one of the two boxes without being seen by the two players (see Figure 1 for a depiction of the experimental setup). The game consists of guessing where the game-master has hidden the doll. The game starts with the experimenter explaining verbally to the players the rules, showing them an example of a sequence: “Now you and (the other partner’s name) will play a game together called “guess where it is.” I’ll show you how it is played. Here are two boxes and a little doll. I will hide the doll under one of the two boxes, but you won’t see where I hide it because I will put this paper board in front of you, like this.” After positioning the board, the experimenter moves the boxes around and then removes the board, placing it on the side of the table. The experimenter then informs the partners that they have to guess where the doll has been hidden by pointing at one of the boxes. Next, without revealing the doll’s location, the experimenter asks: “It is all clear?”. If both partners answer positively, then the play started. All children understood the instructions the first time. The children were also informed that they would receive a packet of stickers at the end of the game to thank them for their participation. Once the game began, the experimenter told the child that the partner (referred to by her/his/its name) would always make the first guess. The position of the doll was established *a priori* to correctly instruct (or program, if NAO) the play partner’s choice during each phase of the game.

The TG involves three independent phases. The first phase [Trusting Acquisition (TA)] aims to assess the participant’s acquisition of trust in the other player by calculating how many trials elapse before the child follows the other player’s guess. Trust is assumed when the child follows the other player’s guess on three consecutive trials. After trust acquisition, the game switches into the second phase [Mistrust Acquisition (MA)], which assesses the participant’s acquisition of mistrust in the other player by calculating how many trials it takes for the child not to follow the other player’s guess. Mistrust is assumed when the child does not follow the other player’s guess on three consecutive trials. The last phase [Trusting Restoration (TR)] shares the same play structure as the initial phase. The game lasted, on average, between 10 and 20 min.

Each phase consisted of a maximum of 10 trials and ended after trust acquisition (phase 1), mistrust acquisition (phase 2), and trust restoration (phase 3). The switch to the following phase also occurred if the participant completed 10 trials within a given phase without completing the three-trial sequence. The dependent variable (DV) was the number of trials the child required before acquiring trust or mistrust. For example, in the initial phase, if the child started to follow the other player for three consecutive trials after the second trial (i.e. 0 0 1 1 1), the participant scored 2. If the child displayed trust immediately (i.e. 1 1 1), s/he scored 0. If the child completed the 10 trials within each phase without acquiring trust or mistrust, she/he scored 8, which is the maximum value that could possibly be attributed before ending the phase with a three-trial sequence. To compare data in the analyses, trust and trust restoration indexes were reversed to indicate, alongside trust loss, a comparable measure of the tendency to trust. Thus, a child could score between 0 (low trust) and 8 (high trust).

For the treatment of missing cases, we considered mean, median, and mode values, as well as children’s most common response patterns. The median was ultimately chosen as the most representative index for replacing missing values. Accordingly, two children were recovered for age groups 3, 5, and 9 years; one child was recovered for the age group 7 years. When an entire session was missing, the values were *not* replaced and the child was removed from the analyses. Accordingly, one child was removed from age group 3 years and three children were removed from age group 7 years.

#### Theory of Mind

The Unexpected Transfer task (Wimmer and Perner, 1983) and the Unexpected Content task (Perner and Wimmer, 1985) were used to evaluate first-order ToM by assessing the acquisition of false beliefs understanding. First-order ToM entails a recursive thinking, which implies the meta-representation or the representation of a mental representation of a low complexity level, of the kind “I think that you think…”. Children exhibit this competence at around 4 years of age with the emergence of false beliefs. The child is told a story involving two doll characters. One of the characters is deceived with respect to either the location or contents of an object and the child is tested for his/her ability to understand the character’s false belief. For example, the unexpected transfer story is about two siblings playing with a ball in a room. One of the children puts the ball in a box and leaves the room. Meanwhile, the other child takes the ball out of the box, puts it in the basket and goes away. Finally, the first character comes back in the room and wants to play with the ball. At the end of the story, the experimenter asks the child the following questions: “What is the first place where she will look for the ball?”—referring to the first character (first-order false belief question); “Where did the child put the ball before going away?” (control memory question); “Where really is the ball?” (reality control question). The answers to the two control questions (memory and reality) were used to filter the children’s performance. Having passed control questions, the test question about false belief is scored 1 if correct and 0 if incorrect.

The development of a second-order false belief competence was assessed through the Ice-Cream Van task (Perner and Wimmer, 1985) and the Look-Prediction task (Liverta Sempio et al., 2001; Astington et al., 2002). Second-order ToM implies a meta-representation of a greater complexity with respect to first-order ToM, of the kind “I think that you think that s/he thinks…”. Children aged from 7 years have typically matured this competence, although it can also emerge at an earlier age. The second-order ToM stories involve three characters presented on a storyboard. For example, the ice-cream van story is about Maria and Giovanni, who – while playing in the park – see an ice-cream van. Maria wants to buy an ice cream, but she has no money. She therefore decides to go home to take the money, sure that the ice-cream van will stay in the park. However, while Maria is away, Giovanni sees the ice-cream van moving away. Giovanni asks the ice-cream man where he is going, and the ice-cream man replies that he is going in front of the school to sell more ice creams. While Maria is leaving home, she sees the ice-cream man and she asks him where he is going. After knowing that he is moving to school, she says that now that she has the money, she can follow him to school. At the end of the story Giovanni goes to Maria’s house, and asks her mother where her friend is. Maria’s mum answers that Maria has just gone out to buy an ice cream. The child (participant) is then asked the following questions: “Where does Giovanni think Maria went to buy the ice cream? (second-order false belief); “Why does Giovanni think so?” (justification); “Does Maria know that the ice-cream van is in front of the school?” (first-order false belief); “Does Giovanni know that the ice-cream man spoke with Maria while she was leaving her house?” (reality control question); “Where did Maria go to buy the ice cream?” (memory control question). For both second-order false belief tasks, having passed the control questions, children scored 1 for correct statements and 0 for incorrect statements on both test and justification questions. A second-order false belief task total score was then calculated ranging from 0 (no response) to 2 (completely correct response) (Perner and Wimmer, 1985).

#### Separation Anxiety Test–Family Version (F-SAT)

The Separation Anxiety Test is a semi-projective task that evaluates the child’s mental representation of his/her attachment to the caregiver. The original version developed by Hansburg (1972) for adolescents was adapted by Klagsbrun and Bowlby (1976) for children aged 4 to 7 years. In the latter version, six pictures are presented to the child, each depicting a situation of separation from a familiar caregiver. The child is asked to describe the protagonist’s feelings, to justify them, and to predict what the protagonist will do, thereby probing the coping strategy of the protagonist. The Italian version used in this study (Liverta Sempio et al., 2001) is based on a modification of other versions of the same task (Fonagy et al., 1997).

The coding reflects three dimensions: (1) attachment, i.e. the ability to express vulnerability and need; (2) self-confidence, i.e. the ability to autonomously face separation; and (3) avoidance, i.e. the propensity to speak about the separation. Participants score 1 for each dimension. The final score is the result of the sum of the scores in the attachment scale and in the self-confidence scale, and of the sum of the inverse of the avoidance scale, calculated by subtracting this score from the total amount potentially obtainable on this scale. Scores range from 6 to 36, with higher scores reflecting greater quality of attachment relationship.

#### Executive Function Skills

Children aged 3 and 5 years were administered the DCCS assessing the capacity to switch responses [for a full description of the test, please refer to Zelazo (2006)]. Seven and 9-year-olds’ executive functions were assessed using “A Developmental NEuroPSYchological Assessment” subtest (NEPSY II; Korkman et al., 2007), testing the ability to inhibit automatic responses and to switch between response types. The child looks at a series of black and white shapes or arrows and names either the shape or direction or makes an alternate response, depending on the color of the shape or arrow. In the present study, we used the combined scores of the Inhibition NEPSY-II subtest, which associates accuracy and speed of response. For a detailed description of the scoring criteria, please refer to the manual (Korkman et al., 2007).

### Experimental Procedure

#### Introducing the Play Partners

On a day that preceded the main experimental session, children were introduced to three play partners (two humans – a boy and a girl – and the robot) through video clips displayed in class on a large projector. In the videos, each of the potential partners said the same sentence: “Hello, my name is. I will be playing with you in the next days. See you soon. Bye.” The videos represented the actors while exiting a room and waving their hand to say goodbye. In this way, the children saw that the robot NAO could walk, talk, and move its head and arms.

#### Experimental Sessions

The children were tested individually in a quiet room in their kindergarten or school. Tests were carried out by two researchers both in the morning and in the afternoon during normal activity. In the first session, the administration of the battery lasted approximately 20–30 min, depending on the child’s age. The administration of the task in the second session took about 35–45 min.

The first session started with the administration of AMS. The five AMS state categories (Perceptive, Emotional, Desires and Intentions, Imaginative, and Epistemic) were randomized across children. Afterward, children participated in the TG. At this point, the partner (i.e. human or robot) entered the experimental room and was introduced by the experimenter by his/her name: “Do you remember, this is …”. Then, both the child and the partner were invited to sit on the ground on a plastic carpet in front of an *ad hoc* table. The plastic carpet was used to correctly position NAO when children interacted with the robot. A paper black board was positioned next to the table, and was used to cover the setting when playing. A female experimenter played with girls and a male experimenter played with boys. Half of the children played with the robot in the first session and with the human in the second session. The other half underwent the reversed play order.

After the game, the child was administered one of the two first-order ToM tasks and, starting from 5 years of age upward, one of the two second-order ToM tasks. The order of the ToM tasks was randomized across children, so that those who performed, for example the unexpected transfer task in the first session, completed the unexpected content task in the second session. The same was true for the second-order ToM tasks. Finally, children were given two further assessments: SAT and executive function.

### Statistical Analysis

Statistical analysis was carried out using the IBM Statistical Software Platform SPSS (v. 19.0). To evaluate possible differences in children’s tendency to trust the human and robot partner as a function of the child’s age and trust phase (acquisition, loss, and restoration), a repeated measures General Linear Model (GLM) analysis was carried out. The DV was the number of trials until children followed their partner (trust acquisition), stopped following their partner (trust loss), and again followed their partner (trust restoration) during the TG. To compare data from the three phases, trust and trust restoration indexes were reversed to indicate, together with trust loss, a comparable measure of the *tendency to trust*. That is, for all three phases of the TG, greater numbers correspond to a greater tendency to trust.

Additionally, correlation analyses (Pearson’s *r*) were carried out to evaluate the relationship between the tendency to trust and (1) the quality of attachment relationships (SAT), (2) ToM (first- and second-order false beliefs tasks), and (3) executive function skills.

Finally, to assess possible differences in children’s mental states attribution to the robot with respect to the human partner, a repeated measures GLM analysis comparing AMS scores between human and robot was carried out as a function of the children’s age. For all the GLM analyses, the Greenhouse–Geisser correction was used for violations of Mauchly’s Test of Sphericity, *P* < 0.05. All *post hoc* comparisons were Bonferroni corrected.

## Results

### Trusting Game

The GLM analysis, with three levels of *phase* (acquisition, loss, and restoration) and two levels of *agency* (HB and RB) as within-subjects factors, and *age group* (four levels) as the between-subjects factor (3, 5, 7, and 9 years), was carried out to assess children’s tendency to trust in the human and in the robot. An inspection of the box plots displaying the performance of each age group showed no extreme cases.

**FIGURE 2:**
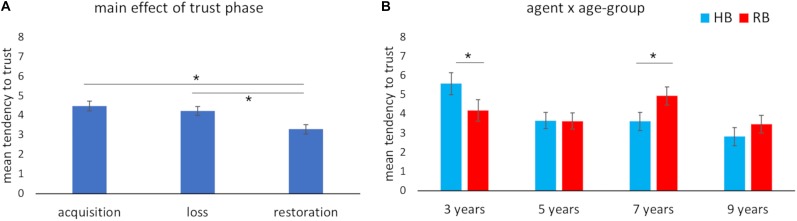
Trust scores for the Trusting Game. Children’s average tendency to trust during the Trusting Game **(A)** across the three phases of trust (acquisition, loss, and restoration) and **(B)** across age groups for the human (HB) and the robot (RB) when controlling for the effect of Theory of Mind. The bars represent the standard error of the mean. *indicates significant differences.

The results revealed a main effect of *phase* (Figure 2A), *F*(2, 172) = 10.51, *P* < 0.001, partial-η^2^ = 0.11, δ = 0.99, indicating that, independent of agency and age group, children exhibited a lower tendency to trust in phase 3 (trust restoration), compared to both phase 1 (trust acquisition), *M*diff = 1.19; SE, 0.26; *P* < 0.001, and phase 2 (trust loss), *M*diff = 0.94; SE, 0.27; *P* < 0.01. Additionally, age-related differences were found, *F*(3, 86) = 8.76, *P* < 0.001, partial-η^2^ = 0.23, δ = 0.99. More specifically, 3-year-olds showed a greater tendency to trust than the other age groups including the 5-year-olds, *M*diff = 1.83; SE, 0.52; *P* < 0.01; 7-year-olds, *M*diff = 1.1; SE, 0.53; *P* < 0.05; and 9-year-olds, *M*diff = 2.64; SE, 0.53; *P* < 0.001. No interactions were found between *phase* and *age group*, *P* > *0.05*. Additionally, *agency* did not have any impact as a main effect and in the interaction with the other variables, *P* > *0.05*.

Having found a consistent correlation across ages between first-order ToM and performance in the TG as described below, a further GLM was carried out using first-order ToM as a covariate. This analysis revealed a main effect of *agency* (Robot – RB > Human Being – HB), *F*(1,85) = 4.99, *P* < 0.05, partial-η^2^ = 0.06, δ = 0.60, and a significant interaction of *agency* × *age group, F*(3, 172) = 2.81, *P* < 0.05, partial-η^2^ = 0.09, δ = 0.66. The *post hoc* analyses showed that while 3-year-olds tended to generally trust in the human more than in the robot, *M*diff = 1.04; SE, 0.67; *P* < 0.05, children aged 7 years tended to trust in the robot more than in the human, *M*diff = 1.33; SE, 0.56; *P* < 0.05. This interaction is plotted in Figure 2B.

### Correlations

#### Trusting and SAT

As shown in Table 1, most of the significant correlations between the quality of attachment relationships (SAT) and the tendency to trust were positive, i.e. more securely attached children showed a greater tendency to trust in the play partner’s choice. These relationships were found mainly when children played with the human, and especially in the youngest age group. For 3-year-olds, all SAT dimensions (except for avoidance) correlated positively with a greater tendency to trust in the human, including during the trust loss phase. For 7-year-olds, quality of attachment positively correlated with the tendency to trust during the trust loss phase.

**TABLE 1:** Association between Trust and SAT.

		**SAT sub-dimensions**							
		**Playing with human**				**Playing with robot**			
**Trust phase**	**Age group (*N*)**	**Attachment**	**Self-confidence**	**Avoidance**	**TOT**	**Attachment**	**Self-confidence**	**Avoidance**	**TOT**
**(A)**	3 years (17)	0.429	0.606**	–0.236	0.422	0.161	–0.053	0.14	–0.132
Acquisition	5 years (20)	–0.042	–0.227	0.088	–0.205	0.282	0.007	–0.004	0.1
	7 years (22)	0.2	0.008	–0.264	0.302	–0.135	0.063	–0.12	–0.06
	9 years (23)	0.477*	–0.118	–0.306	0.259	0.412	–0.003	0.056	0.224
	Overall	–0.059	0.027	0.156	–0.172	0.097	–0.025	0.102	–0.056
**(B)**	3 years (17)	0.557*	0.746**	–0.248	0.579*	0.393	0.353	–0.267	0.272
Loss	5 years (20)	–0.195	–0.012	0.023	–0.095	0.198	–0.025	0.138	–0.014
	7 years (22)	0.526*	0.066	0.068	0.412	0.188	0.153	0.247	–0.021
	9 years (23)	–0.177	–0.273	0.487*	–0.262	0.048	0.209	–0.106	0.187
	Overall	–0.146	0.092	0.218*	–0.14	0.07	0.158	0.052	0.007
**(C)**	3 years (17)	0.298	0.418	0.039	0.163	0.629**	0.459	–0.307	0.48
Restoration	5 years (20)	0.129	0.093	–0.147	0.264	0.362	0.079	–0.118	0.33
	7 years (22)	–0.17	0.044	0.05	–0.106	0.269	0.22	–0.016	0.139
	9 years (23)	–0.146	–0.073	0.068	–0.188	0.077	0.009	0.109	0.084
	Overall	–0.066	0.128	0.05	0.005	0.203	0.205	–0.08	0.209

Correlations between the tendency to trust during **(A)** the acquisition, **(B)** loss, and **(C)** restoration of trust and the quality of attachment relationships (SAT), sub-dimensions (attachment, self-confidence, avoidance), as well as total SAT. **Correlation is significant at the level 0.01 (two-tailed). *Correlation is significant at the level 0.05 (two-tailed). Significant values are in bold.

For 9-year-olds, the results also showed a positive relationship between trust acquisition and the SAT sub-dimension of attachment, indicating that more securely attached children tended to acquire trust quicker. Additionally, among 9-year-olds, there was a positive correlation between the tendency to trust during the trust loss phase and the SAT sub-dimension of avoidance. This correlation was also significant across ages.

A positive correlation was finally found between the SAT sub-dimension of attachment and the tendency to trust in the *robot* for 3-year-olds during the restoration of trust phase. No significant correlations were found for 5-year-olds.

#### Trusting and ToM

The scores on the two ToM tasks were merged into one single score for each level of complexity (first and second order). A low level of ToM performance (coded 0) included children who scored 0 (failed) on both tasks, whereas a high level of performance (coded 1) included children who passed at least one ToM task at each complexity level. Table 2 reports descriptive data for the ToM tasks.

**TABLE 2:** ToM descriptives.

**Age group (years/*N*)**	**First-order ToM**		**Second-order ToM**	
	**Low (%)**	**High (%)**	**Low (%)**	**High (%)**
3 (22)	68	32	–	–
5 (24)	25	75	50	50
7 (24)*	0	96	20	76
9 (23)	0	100	13	87

Percentage of children in each age group (years) displaying a low or high first and second-order Theory of Mind (ToM). *One missing case.

All correlations found between the tendency to trust and ToM scores were negative. Thus, greater ToM abilities were associated with a lower tendency to trust, i.e. with a more reflective tendency to trust. This relationship was independent of the partner’s agency (human or robot) or the child’s age. The tendency to trust was often significantly correlated with first-order ToM, which was therefore included as a covariate in the GLM model described above. Finally, a substantial negative correlation between the tendency to trust and second-order ToM was observed during the acquisition of trust for children aged 7 years when playing with the human. These statistics are reported in Table 3.

**TABLE 3:** Association between Trust and ToM.

		**Playing with the human**		**Playing with the robot**	
**Trust phase**	**Age group (*N*)**	**First order**	**Second order**	**First order**	**Second order**
1 – Acquisition	3 years (21)	–0.225	−	–0.306	−
	5 years (24)	0.091	0.071	–0.231	–0.187
	7 years (23)		−0.501*		0.029
	9 years (23)		–0.176		–0.03
	Overall (90/69)	−0.315**	–0.235	−0.278**	–0.144
2 – Loss	3 years (21)	–0.28	−	−0.424*	−
	5 years (24)	0.079	–0.356	–0.303	–0.285
	7 years (23)		–0.267		0.004
	9 years (23)		–0.091		0.112
	Overall (90/69)	−0.278**	−0.244*	−0.365**	–0.09
3 – Restoration	3 years (21)	–0.119	−	–0.329	−
	5 years (24)	0.024	–0.151	–0.033	0.058
	7 years (23)		0.358		0.317
	9 years (23)		–0.298		–0.019
	Overall (91/70)	–0.163	–0.066	−0.219*	0.143

Correlations between the tendency to trust and ToM score. **Correlation is significant at the level 0.01 (two-tailed). *Correlation is significant at the level 0.05 (two-tailed). First-order ToM had a constant value for age groups 7 and 9 years and correlations could not be calculated. Significant values are in bold.

#### Trusting and Executive Function Skills

Children aged 3 and 5 years were administered the DCCS, which assesses the capacity to switch between responses (Zelazo, 2006). The same skill was assessed in 7- and 9-year-olds using the “Developmental NEuroPSYchological Assessment” subtest (NEPSY II; Korkman et al., 2007). To compare data across age groups, scores were standardized.

Significant age-related positive relationships were found between the ability to switch and the tendency to trust during the restoration phase among 3-year-olds when playing with the human, *r*(19) = 0.49, *P* < 0.05, and among 7-year-olds when playing with the robot, *r*(22) = 0.43, *P* < 0.05.

### Attribution of Mental States

**FIGURE 3:**
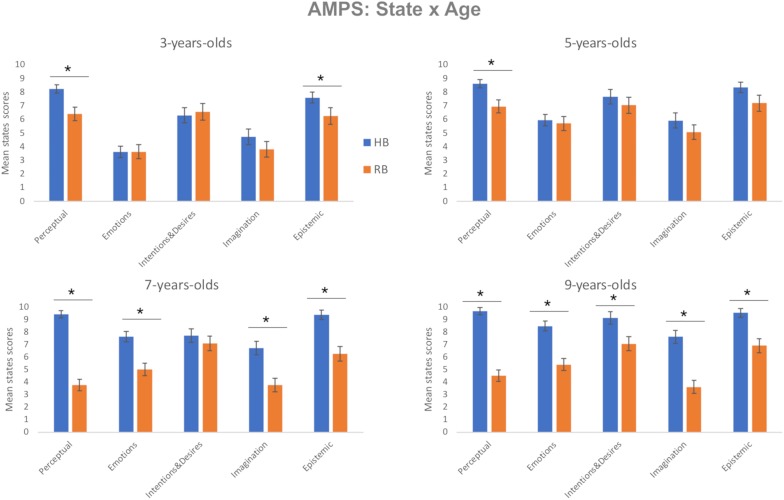
Children’s scores on the Attribution of Mental States (AMS) scale. AMS mean scores for the human (HB = blue bar) and the robot (RB = orange bar) for each age group (3-, 5-, 7-, and 9-year-olds) as a function of state (Perceptual, Emotions, Intentions and Desires, Imagination, Epistemic). The bars represent the standard error of the mean. * indicates significant differences.

A repeated measures GLM analysis comparing AMS scores between human and robot, with five levels of *state* (perceptual, emotions, intentions and desires, imagination, and epistemic) and two levels of *agency* (HB and RB) as within-subjects factors, and *age group* (four levels) as the between-subjects factor, showed a main effect of *state*, *F*(4, 332) = 71.72, *P* < 0.001, partial-η^2^ = 0.46, δ = 1, a main effect of *agency* (HB > RB), *F*(1, 83) = 82.10, *P* < 0.001, partial-η^2^ = 0.50, δ = 1, an interaction of *state* × *age group, F*(12, 332) = 5.18, *P* < 0.001, partial-η^2^ = 0.16, δ = 1, an interaction of *agency* × *age group, F*(3, 83) = 8.66, *P* < 0.001, partial-η^2^ = 0.24, δ = 0.99, an interaction of *state* × *agency, F*(12, 332) = 19.99, *P* < 0.001, partial-η^2^ = 0.19, δ = 1, and a three-way interaction between *state, agency*, and *age group*, *F*(12, 332) = 2.31, *P* < 0.01, partial-η^2^ = 0.85, δ = 0.96. This interaction is represented in Figure 3.

Exploring the three-way interaction, the most consistent difference was for the attribution of perception (HB > RB), which was significant for all four age groups, *P* < 0.01. Attribution of epistemic states was also greater for HB than RB for all age groups, *P* < 0.01, except 5-year-olds, for whom there was a trend toward significance, *P* = 0.07. Attributions of emotion and imagination were similar for HB and RB among 3- and 5-year-olds, but greater for HB among 7- and 9-year-olds, *P* < 0.05. Finally, only 9-year-olds ascribed greater intentions and desires to HB than RB, *M*diff = 4.00; SE, 0.63; *P* < 0.001. These *post hoc* analyses are summarized in Figure 3. Overall, these analyses confirm that humans and robots are differentiated even by 3-year-olds with respect to perception and epistemic states with that differentiation spreading to all five states among 9-year-olds.

## Discussion

The present study investigated trust dynamics when children aged 3, 5, 7, and 9 years played a TG *in vivo* with either a human or a robot partner. Children’s tendency to trust decreased across the three phases of the game, from acquisition to restoration of trust. Also, 3-year-olds displayed a greater tendency to trust in both play partners compared to the other age groups, although initially placing their trust more easily in the human than in the robot. The opposite was observed for the 7-year-olds, who generally placed more trust in the robot than the human.

To better understand age changes in trust, the results for quality of attachment relationships, false belief understanding, and executive function skills were examined. It has been previously shown that children aged 3 and 4 years are likely to endorse information provided by someone who proved accurate in the past (see also Koenig and Harris, 2005; Pasquini et al., 2007; Harris, 2007). The results for SAT deepen this observation. Among 3-year-olds, we found that the SAT sub-dimension of self-confidence was positively associated with selective trust in the human, probably because these children’s past relationships were secure, thus increasing the perception of the unfamiliar experimenter as trustworthy. On the contrary, the robot was an entity with which children had never had any relational experience, further skewing the youngest children’s trust preference toward the human. Additionally, it was found that the youngest children – and particularly securely attached children – showed a tendency to retain trust during the loss of trust phase, confirming a certain behavioral rigidity as introduced above. However, when realizing that the other was no longer trustworthy, they switched to trusting the robot more. This result supports the observation that when very young children’s expectations are betrayed (loss of trust), they are less forgiving than older children (Harris, 2007); additionally, our findings enrich previous results (Corriveau and Harris, 2009) by further showing that children who are securely attached in infancy are more flexible when investing their trust.

The development of a fundamental cognitive ability makes a substantial contribution to trust dynamics in child–robot interaction across all age groups. According to our findings, the development of ToM appears to temper the relation between quality of attachment relationships and trust, by introducing into the trust matrix a mentalistic evaluation of the other’s judgment based on an awareness of her/his/its beliefs. More specifically, children who had developed at least first-order ToM also knew that the other player did not know the position of the doll, and was therefore an unreliable informant. Not by chance, the effect of ToM on trusting behavior was most evident at 3 and 7 years of age, typically marked by the development of increasingly complex levels of ToM. When children start developing the concept of the other’s mind, they are also able to evaluate whether the other (either a human or a robot) is trustworthy on the basis of informational access. Preferential trust in either agent then moderates.

The dichotomy found between the younger and older age groups in the AMS to the robot and the human further helps to delineate the children’s perception of the robot as a mentalistic agent: For younger children, the robot is perceived as more mentalistically comparable to the human than for older children. Nevertheless, when younger children decided to trust a play partner, the affective component prevailed over the more “cool” mentalistic component, defining the preferred relational target accordingly (i.e. the human). On the other hand, the trust attributed to the robot by older children may stem from the dominance of a cognitive over an affective engagement.

In support of agent-specific differences in trusting behavior between 3- and 7-year-olds, the results also revealed a positive association between the ability to switch and trusting behavior during the trust restoration phase among children aged 3 and 7 years. Strikingly, and consistent with the data discussed above, these relations were specific to playing with the human partner for the 3-year-olds, and to playing with the robot for the 7-year-olds. In general, these correlations indicate that a greater tendency to recover trust in the other is associated with the development of the ability to switch. The specificity related to the play partner’s agency further underlines the relevance of the interactive partner for the child and reflects children’s engagement with one or the other player: 3-year-olds’ selective trust in the human was plausibly influenced by the quality of attachment relationships – as also evidenced by data on attachment described above; 7-year-olds preferential trust in the robot was possibly due to an emerging familiarity with artificial devices typical of this age. These results shed light on previous findings (Lucas et al., 2013) that showed that, compared to false belief understanding, executive functions skills do not play an essential role in building selective trust in an informant, at least within specific cultural frames. In our study, we did find that executive function skills played a role, though not during the phase of trust acquisition, but rather during trust restoration. Here, the child’s ability to switch was possibly required to re-organize information and re-establish trust in an informant that, during the loss of trus phase, became unreliable. Executive function skills may then be specifically involved in building trust only under specific conditions, which had not been empirically assessed so far.

## Conclusion, Limitations, and Future Directions

The present study provided some insight into the dynamics of trust both when relating to a human and a robot partner. Our results highlighted the impact of cognitive development, as well as children’s attachment history. We found that cognition and attachment operated separately (given the absence of a direct correlation between these two dimensions) on the establishment of trust. Particularly for children aged 3 years, trust appears to be significantly influenced by the affective dimension of trust, especially when interacting with a human. Interestingly, although securely attached children exhibited a greater tendency to trust the human, they also shifted their trust more rapidly in the trust restoration phase with the robot. This may be due to the lack of any affective bond with the robot and to the child’s cool relational attitude toward it. Effectively, this would render the robot a more “forgivable” partner.

Also, the development of false belief understanding proved to play a significant role in the establishment of trusting relationships. In particular, the development of mentalizing abilities enabled children to reflect rationally on the fact that the other player had exactly the same guessing opportunities as they did, and was therefore as susceptible to making mistakes as they were. This moderated the effect of the affective component of trust.

In the present study, the robot proved to be less susceptible to the dynamics associated with the quality of attachment relationships, and thus became a more stable trusted partner. For this reason, and particularly for children with fragile affective relational histories who have difficulties with trust, the robot might fulfill a significant scaffolding role in human–human interaction. However, an evolution of the robot as a social partner is also to be expected. Therefore, different relational dynamics may be anticipated, according to which, perhaps, an affective relation history will be created with this new entity. In this respect, a longitudinal study would further delineate the development of trust in the robot increasing the robustness of the findings. Also, a larger sample size would eventually confirm the observed tendencies.

Last, but not least, the findings from this study may inform disciplines such as Developmental Robotics on how cognitive architectures can be modeled in the robot so as to make it trusting in the human partner in a “human-like” fashion, as discussed above. This circular behavior would make the human–robot relationship increasingly ecological and, ultimately, trustful. Starting, for example, from the architectural model designed by Vinanzi et al. (2019), in which the robots’ trust in an informant varied as a function ToM, the present findings clearly indicate further psychological factors that may be integrated in the robot to design the *robot’s trust in the human* at different developmental levels. Recent technical and theoretical achievements in the field of social robotics have encouraged researchers to develop social robots as tutors and learning companions for children (e.g. Movellan et al., 2009; Tanaka and Matsuzoe, 2012; Breazeal et al., 2016). Therefore, studying the mechanisms by which children learn from robots, and vice versa, is of vital importance.

## Data Availability Statement

All data needed to evaluate the conclusions in the article are present in the article.

## Ethics Statement

The studies involving human participants were reviewed and approved by the Ethic Committee, Università Cattolica del Sacro Cuore, Milano, Italy. Written informed consent to participate in this study was provided by the participants’ legal guardian/next of kin.

## Author Contributions

AM, AC, PH, DM, CD, and FM conceived and designed the experiment. FM and GP conducted the experiments in schools. AM and FM secured ethical approval. CD and DM carried out the statistical analyses. AC granted the use of the SoftBank Robotics NAO humanoid robot. All authors contributed to the writing of the manuscript.

## Conflict of Interest

The authors declare that the research was conducted in the absence of any commercial or financial relationships that could be construed as a potential conflict of interest.
